# COVID-19 has exposed the need for health system assessments to be more child health-sensitive

**DOI:** 10.7189/jogh.12.03048

**Published:** 2022-07-16

**Authors:** Danielle EMC Jansen, Susanne Carai, Eileen Scott, Cassandra Butu, Ioana Pop, Minhye Park, Dheepa Rajan, Martin W Weber, Ingrid Wolfe

**Affiliations:** 1 Department of General Practice & Elderly Care Medicine, University Medical Centre Groningen, University of Groningen, Groningen, the Netherlands; 2 Department of Sociology and Interuniversity Centre for Social Science Theory and Methodology (ICS), University of Groningen, Groningen, the Netherlands; 3 Accare, University Centre for Child and Adolescent Psychiatry, Groningen, the Netherlands; 4 WHO Athens Office for Quality of Care and Patient Safety, Athens, Greece; 5 Universitat Witten/Herdecke, Witten, Germany; 6 Public Health Scotland, Edinburgh, Scotland; 7 WHO Country Office, Bucharest, Romania; 8 WHO, Department for Health System Governance and Financing, Geneva, Switzerland; 9 Department of Women's and Children's Health, King's College London, London, England

The COVID-19 pandemic is directly and indirectly impacting children’s health [1]. Although the evidence base in this area is expanding, there is still much we do not know. Paediatric Multisystem Inflammatory Syndrome temporally associated with COVID-19 (MIS-C: Multisystem Inflammatory Syndrome – Children) and long COVID are the most notable direct effects of COVID-19 infection on children [2,3], though they are rare [4]. Data suggest that children represent a very low proportion (between 1 and 3% of the total) among known COVID-19 infections, at least in the early stages of the COVID-19 pandemic [5]. However, because children are likely to have a higher proportion of asymptomatic infection than adults [6], this might be an underestimation. The most significant population-level health harms for children from the pandemic are indirect, reflecting the impact of health system adaptations and policy changes [1,7] such as elective care postponement and cancellation and possibly remote (instead of face-to-face) consultation which remains unevaluated. Evidence is emerging from a variety of different countries, illustrating the breadth of problems. In England, a large national longitudinal study found an increase in the rates of probable mental health disorders among children aged 5-16 before (10.8% in 2017) and after (16% in 2020) the pandemic. However, more than a fifth of children and 44.6% of 17-22-year-olds with a probable mental health disorder did not seek mental health service support due to the pandemic [8]. Similarly, a very stark drop in accident and emergency attendances for mental health problems during and after the first COVID-19 policy measures and a steep rise in the first months of 2021 were reported in Scotland [9]. Emergency Department contacts among children in England fell to 40% below usual levels during the peaks of the pandemic, affecting all types of presentation, ranges of acuity, and age groups [9]. In Romania, hospitalization rates of children fell by 70% since the beginning of the pandemic and have not reached pre-pandemic levels [10]. It is unknown if this drop is affected by limited access due to COVID restrictions or by other factors, such as a lower infection rate due to social distancing. The rapid and necessary scaling down of health care to redeploy paediatric and child health staff to adult services [11,12] may have led to dangerous disruptions to the delivery of timely and high-quality health care for children, especially for those with chronic illness, disabilities and for those already experiencing barriers in access to care before COVID-19, such as minority ethnic groups including Roma and Travellers populations. Notably, there is no ethical framework for decision-making that considers children’s rights in such situations. Importantly, health system changes resulting from the pandemic also include creative innovations such as new ways of delivering outpatient services and the widespread adoption of technological solutions for delivering remote consultations which may enable families to access the health care they need [13]. These innovations must be urgently evaluated for impact on health and equity of care, especially for specific populations with distinct needs such as neurodivergent children, vulnerable children, and children with communication needs.

Health system shocks such as those related to the COVID-19 pandemic affect governance and finance systems, technology, workforce, and of course health care services. The specific effects of a health system shock on children and young people, as populations with specific needs, can be easily overlooked in general health system assessments that are not designed to be sensitive to child health. In this viewpoint, we argue for more child health sensitivity, including more child health metrics, in health system assessments and performance assessments.

## THE VALUE AND APPLICATION OF CURRENT HEALTH SYSTEM PERFORMANCE FRAMEWORKS

Well-performing health systems provide a potentially essential contribution to tackling socially patterned inequalities in health and improving health outcomes [14]. The distruptions caused by COVID-19 highlight the urgent need for building strong and resilient health systems that can effectively absorb shocks by maintaining core functions and, crucially, learn from those shocks [15] to improve.

These distruptions also show the need for reliable and valid indicators for assessing and comparing health system performance within and across European countries. A health system performance framework is essential to understanding and improving performance and learning from crises. It can provide a useful and rich source of evidence for policymakers in decision-making and in tracking progress in meeting key strategic national health goals. Health system performance assessment and comparative studies have been increasing since the World Health Report in 2000 [16] and methods have been developed to suit the complexities of health systems research [17]. However, there has been less focus on children in health systems, particularly among high income countries with less pronounced problems than among low- and middle-income countries, but where important questions about disparities in high level outcomes such as child survival remain unanswered [18-20].

**Figure:**
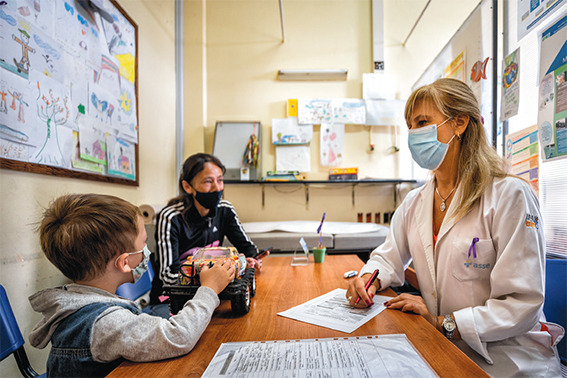
Photo: Source: ©WHO/Blink Media – Tali Kimelman (used with permission).

Several health system performance frameworks for the general population have been proposed, discussed, and adapted at international and national levels [21,22]. Most health system performance frameworks include structures (eg, availability of policies, resources, and capacity for timely access to care) and processes (eg, referral processes, treatment decisions, and collaboration between care professionals) presumed to relate to health outcomes. Almost all health system performance frameworks include equity in health care as a key outcome. While the importance of a health system performance framework is not disputed, their usage presents several challenges. One of the reasons for the multitude of health system performance frameworks lies in the complexities of component parts and their interactions, reflecting the specific needs of children and young people.

## HOW HAS COVID-19 EXPOSED THE NEED FOR HEALTH SYSTEMS TO BE MORE CHILD HEALTH-SENSITIVE?

COVID-19 has emphasized several reasons why adult-focused health system frameworks may omit performance aspects related to children’s health. Children’s health care differs in many aspects from the rest of the population. First, it is family-oriented and often relies on parental involvement in health care decision-making and treatment for their children. This might affect the autonomy of adolescents. The H2020-MOCHA (Models of Child Health Appraised) study [23] found that adolescent autonomy in decision-making is of utmost importance in realising positive outcomes, but sometimes goes against the wishes, beliefs, and interests of parents and of health professionals. This was the case when complying with COVID-19 public health measures (eg, masks) and in decision-making about vaccinating young people against COVID-19 [24]. This added dimension influences care provision and outcomes and must be incorporated into health system performance frameworks to reflect these differences. Furthermore, issues of privacy for children and young people have been highlighted by COVID-19. Adolescents who could not physically visit their health care professional during COVID-19 were forced to consult their professional via eg, telephone calls that placed them in a situation with less to no privacy [25]. Moreover, specific elements of care for children with complex health care needs are not taken into account explicitly by the current health system performance frameworks. For example, the health care and other professionals that children visit change during different stages of their lives, eg, during the transition from child to adolescent and adult services. Brenner et al. [26] showed that none of the 30 European countries involved in their study collected experiences regarding transition from paediatric to adult services from adolescents with complex care needs. Additionally, the vast majority reported no policies or procedures to ensure continuity of care when transitioning to adult services. COVID-19 threatens the continuity and quality of care for this group of children. The complex care of these patients requires a carefully organised and coordinated multidisciplinary team. With COVID-19, in-person, family-centered care provided by a multidisciplinary team has been transformed to “virtual rounds” which are nowhere near equaling the quality of in-person consultations [27]. Health system performance measures that enable the delivery of safe and optimal care are urgently needed [26].

Adult focused health system frameworks may downplay the interfaces with non-health system determinants vital for child health. The ways in which health care for children aligns with public health and universal services for early childhood development, coordinates with children’s social care and education, and cooperates with services for parents and parenting must be considered when assessing how health systems perform and contribute to child health outcomes. In the Netherlands, youth care is an essential service for young people and is considered as social care rather than health care, though they interact closely. Similarly, community health care is a vital component of children’s health care in settings such as the UK, but may fall outside core primary and secondary care services from an adult health system perspective. As never before, COVID-19 demands rapid, constructive and collaborative responses for deliveirng high quality integrated care for children. However, the fragmentation in health and social care systems in many settings impedes such a response [28].

Looking beyond health services, a child-specific health system performance framework must adequately reflect the health system components that underpin health care: governance, financing, analytics, technology, and workforce. Examining just the aspect of governance demonstrates the complexities and differences between children and adults regarding health system performance. A 2017 analysis of governance concerning health systems for children in England found deficits in each aspect of the Transparency, Accountability, Participation, Integrity, and Capacity (TAPIC) framework (a framework consisting of five components of governance that can affect the success or failure of policies), illustrating the contrasts between children and adults [29]. There was an evident lack of policy capacity (the ability to translate knowledge into policy and practice) specific to child health relating to major policy initiatives, including the Health and Social Care Act which completely transformed the NHS in 2012. While there have been signs of improvements in England, notably the NHS Long Term Plan, other recent major policies such as the 2021 NHS data strategy [30] almost completely ignore the distinctive needs of children. Data and analytics are key health system components, providing another example of the differences between adults and children that must be considered in constructing health system performance frameworks. Data from schools, community health, public health, and children’s social care are needed to accurately reflect health system performance for children. Unfortunately, health information systems in many countries are either absent or not designed to provide real-time information for decision-making during COVID-19 times. There is a lack of data standardization and integration between different health information systems, particularly affecting children and adolescents [31].

Finally, children are not part of the economically active population and thus have no say on how resources are spent, nor are their needs reflected in health systems financing measures which reflect the working age population, including children’s parents. Financing of children’s health care is different from adults in many countries. In the Netherlands, according to the Youth Act, care for children with mental health problems is paid by the municipality, while care for adults with mental health problems is paid by the insurer according to the Health Insurance Act. Moreover, children’s status as not economically productive often makes their perception irrelevant to policymakers. Children are usually excluded, for example, from health care satisfaction surveys.

We summarised the main reasons for ensuring child and adolescent-sensitive measures within a health systems performance paradigm. The broad aim of health system assessments is to enable health system strengthening towards improving health system performance and thereby health outcomes. Assessments should provide information on which areas to prioritise and from which examples to learn. The focus for this and related efforts, is directed at the entire population. The question we address here is about refining the focus directly on babies, children, and young people. The health needs of babies, children, and young people often differ from the general adult population, and the health system needs to respond accordingly, requiring specific qualitative and quantitative performance measures.

## RECOMMENDATIONS FOR THE WAY FORWARD: WHAT DO WE SUGGEST?

As outlined in this viewpoint, the COVID-19 pandemic caused major shocks to health systems. All child health-sensitive issues we raised were already present before COVID-19 made its appearance. However, COVID-19 emphasized their importance and seriousness and the need for reliable and valid systematic and child-specific measures, stressed by the widely-documented health system shocks described in this and many other papers. Governments and policymakers are compelled to give more attention to health systems for children and adolescents than ever before as current disruptions reveal that health systems may not adequately have responded to the effects of this pandemic. To fully understand and anticipate the health system shocks caused by COVID-19 and to prepare our health system for new unknown and unexpected developments, we must adapt health systems performance assessment frameworks to appropriately reflect the needs of children and young people. We make specific recommendations towards achieving a framework that is sensitive to the needs of children and young people.

1. We call for the formation of an international benchmarking group, with close collaboration of health system and child health specialists, to work on children’s health systems focusing on three specific goals:
  - to agree on a set of valid comparable child health-sensitive qualitative and quantitative measures which can be included into health system performance assessments;
  - to analyze and interpret health system assessment information and data;
  - to describe an agenda for children’s health systems and policy research.
2. We call on countries to review how current health systems are meeting children’s and adolescents’ needs and evaluate innovations arising from health system shocks.

The availability of universal, valid, and comparable child health indicators for health system performance assessment is limited. A consensus is needed on indicators for child health and health services that supports effective performance assessment and enables comparison between countries to support learning. We need a detailed understanding about how to deliver health care and how to strengthen health systems in ways that meaningfully and consistently improve child health outcomes [32]. This is an urgent and important deficit which needs to be corrected in order to understand suboptimal child health outcomes and unwarranted variations within and between countries. Well-validated universal outcome measures to assess health system functioning are needed to generate data and insights for fostering improvements in preventive and health care services and comparative benchmarking within and between countries. Securing optimal conditions for child survival, health, and development requires a comprehensive approach to securing a strong health system; this starts with a dedicated focus to improve children’s health systems assessment.
